# Combined analysis of the proteome and metabolome provides insight into microRNA-1174 function in *Aedes aegypti* mosquitoes

**DOI:** 10.1186/s13071-023-05859-1

**Published:** 2023-08-09

**Authors:** Yangrui Luo, Dun Liu, Yuanmei Wang, Fan Zhang, Yankun Xu, Qian Pu, Lu Zhao, Tianqi Wei, Ting Fan, Yuqi Lou, Shiping Liu

**Affiliations:** 1 State Key Laboratory of Resource Insects, Southwest University, Beibei, Chongqing, 400716 People’s Republic of China; 2 College of Marine Life Science, Ocean University of China, Qingdao, 266003 Shandong People’s Republic of China

**Keywords:** *Aedes aegypti*, MicroRNA-1174, TMT proteomics, LC–MS/MS non-target metabolomics, Purine nucleoside phosphorylase, RNA interference

## Abstract

**Background:**

Pathogenic viruses can be transmitted by female* Aedes aegypti* (*Ae. aegypti*) mosquitoes during blood-meal acquisition from vertebrates. Silencing of mosquito- and midgut-specific microRNA (miRNA) 1174 (miR-1174) impairs blood intake and increases mortality. Determining the identity of the proteins and metabolites that respond to miR-1174 depletion will increase our understanding of the molecular mechanisms of this miRNA in controlling blood-feeding and nutrient metabolism of mosquitoes.

**Methods:**

Antisense oligonucleotides (antagomirs [Ant]) Ant-1174 and Ant-Ct were injected into female *Ae. aegypti* mosquitoes at 12–20 h posteclosion, and depletion of miR-1174 was confirmed by reverse transcription quantitative real-time PCR (RT-qPCR). Ant-1174-injected and control mosquitoes were collected before the blood meal at 72 h post-injection for tandem mass tag-based proteomic analysis and liquid chromatography-tandom mass spectrometry non-target metabolomic analysis to identify differentially expressed proteins and metabolites, respectively. RNA interference (RNAi) using double-stranded RNA (dsRNA) injection was applied to investigate the biological roles of these differentially expressed genes. The RNAi effect was verified by RT-qPCR and western blotting assays. Triglyceride content and ATP levels were measured using the appropriate assay kits, following the manufacturers’ instructions. Statistical analyses were conducted with GraphPad7 software using the Student’s t-test.

**Results:**

Upon depletion of mosquito- and midgut-specific miR-1174, a total of 383 differentially expressed proteins (DEPs) were identified, among which 258 were upregulated and 125 were downregulated. Functional analysis of these DEPs using Gene Ontology (GO) and Kyoto Encyclopedia of Genes and Genomes (KEGG) enrichment suggested that miR-1174 plays important regulatory roles in amino acid metabolism, nucleotide metabolism, fatty acid metabolism and sugar metabolism pathways. A total of 292 differential metabolites were identified, of which 141 were upregulated and 151 were downregulated. Integrative analysis showed that the associated differential proteins and metabolites were mainly enriched in a variety of metabolic pathways, including glycolysis, citrate cycle, oxidative phosphorylation and amino acid metabolism. Specifically, the gene of one upregulated protein in miR-1174-depleted mosquitoes, purine nucleoside phosphorylase (*PNP*; AAEL002269), was associated with the purine, pyrimidine and niacin-nicotinamide metabolism pathways. *PNP* knockdown seriously inhibited blood digestion and ovary development and increased adult mortality. Mechanically, *PNP* depletion led to a significant downregulation of the vitellogenin gene (*Vg*); in addition, some important genes in the ecdysone signaling and insulin-like peptide signaling pathways related to ovary development were affected.

**Conclusions:**

This study demonstrates differential accumulation of proteins and metabolites in miR-1174-depleted *Ae. aegypti* mosquitoes using proteomic and metabolomic techniques. The results provide functional evidence for the role of the upregulated gene *PNP* in gut physiological activities. Our findings highlight key molecular changes in miR-1174-depleted *Ae. aegypti* mosquitoes and thus provide a basis and novel insights for increased understanding of the molecular mechanism involved in a lineage-specific miRNA in mosquito vectors.

**Graphical Abstract:**

**Figure:**
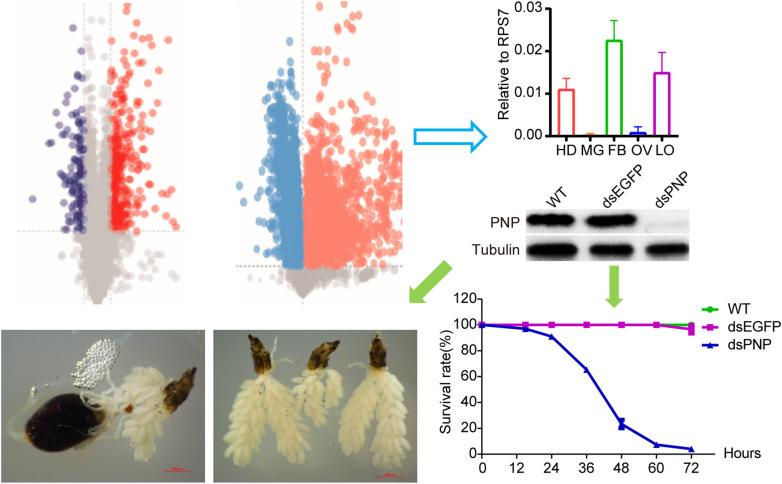

**Supplementary Information:**

The online version contains supplementary material available at 10.1186/s13071-023-05859-1.

## Background

Females of vector mosquito species take blood from vertebrates to obtain the amino acids and other nutrients needed for egg development; consequently, they serve as vectors of a large number of viral pathogens. With global urbanization and the continuous warming of the climate, the risk of mosquito-borne diseases is on the rise worldwide, posing a serious challenge to public health and imposing significant economic burdens on many countries [1]. Over the last few years, a series of promising strategies and technologies for controlling mosquito vectors based on genetic manipulation have been gaining acceptance [2–5]. However, the lack of effective medical interventions still restricts the prevention and control of most mosquito-borne diseases.

Understanding the regulatory pathways in nutrient metabolism of vector mosquitoes is critical for the prevention and control of mosquito-borne viral diseases. In the female mosquito, major pathways in carbohydrate metabolism are synchronized with the high-energy demands of reproduction [6]. The amino acids and proteins in vertebrate blood absorbed by mosquitoes provide the desired nutrients needed for ovary development. Thus, a normal metabolism of amino acids is critical for mosquito reproduction.

In previous studies, we demonstrated that the mosquito- and gut-specific microRNA (miRNA)-1174 (miR-1174) controls sugar utilization, blood absorption, ovary development and egg deposition through its target gene serine hydroxymethyltransferase (*SHMT*) [7]. We also found that knockdown of *SHMT* does not affect mosquito blood absorption, but it does block blood digestion, such that mosquitoes lose the ability to fly, ovary development is stagnant and spawning rate is reduced [8]. The SHMT protein catalyzes the cross-conversion of l-serine and glycine [9, 10]. Purine and pyrimidine biosyntheses are inhibited when SHMT activity is reduced [11]. Although the miR-1174-SHMT axis plays important roles in the gut of mosquitoes, the molecular mechanisms of miR-1174 action in such physiological processes as blood digestion, metabolic homeostasis and reproduction remain largely unknown.

We undertook the present study to decipher the miR-1174-responsive pathways involved in the biological processes of mosquitoes by using proteomics and metabolomics techniques. We analysed the functions of the differentially expressed proteins and metabolites using Gene Ontology (GO) and Kyoto Encyclopedia of Genes and Genomes (KEGG) enrichment. Integration of proteomics and metabolomics was performed to identify the signal pathways for those differentially expressed proteins and metabolites. The biological roles of these miR-1174-responsive genes were investigated by RNA interference (RNAi). Specifically, knockdown of the gene encoding purine nucleoside phosphorylase (PNP) seriously inhibited blood digestion and ovary development and shortened the longevity of adult mosquitoes. Our work further expands and deepens current knowledge on the regulatory mechanism of mosquito-specific miR-1174, and may provide a new target for the development of novel techniques for mosquito control at the molecular level.

## Methods

### Insect breeding and sample collection

*Ae. aegypti* (NIH Rockefeller strain) mosquitoes used in this study were bred as reported previously [8]. Briefly, the *Ae. aegypti* strain colony was reared at 28 °C and 75% relative humidity under a 12/12-h light/dark photoperiod. Larvae were reared in water supplemented with an artificial diet composed of yeast and rat chow (1:1 ratio). A cotton pad soaked in water and a cotton pad soaked in 10% glucose solution were provided for adult mosquitoes before and after a blood meal. Three- to four-day old female mosquitoes were allowed to feed on white rats anesthetized with isoflurane. Laboratory vertebrate animals were maintained in accordance with the guidelines approved by the Southwest University Animal Care and Use Committee. To harvest mosquito individuals and tissues after a blood meal, the blood bolus was removed from the gut by pinching with forceps under a stereomicroscope. Mosquito tissues were dissected in phosphate-buffered saline (PBS). All experiments in this work were performed on adult female mosquitoes only.

### Proteomic analysis

Mosquitoes with or without a blood meal were fed on 10% glucose solution in an artificial climate incubator maintained at 28 °C and 75% relative humidity. Antisense oligonucleotides (antagomirs [Ant]) Ant-1174 and Ant-Ct were purchased from Dharmacon Inc. (Lafayette, CO, USA) and injected into adult female mosquitoes at 12–20 h posteclosion (PE) as described previously [7]. Three biological replicates were set for both the Ant-1174-injected (Ant-1174) and control groups, with 60 female and 30 male mosquitoes in each cage. The Ant-1174-injected females and controls were collected before a blood meal at 72 h post-injection (PIJ), and 20 females randomly selected from each cage were placed in a 1.5-ml centrifuge tube, frozen in liquid nitrogen and stored at − 80 ℃ before use. Six Eppendorf tubes (Eppendorf Co., Eppendorf, Hamburg, Germany) containing samples (Ant-1174: control = 3:3) were sent to Shanghai Biotree Biomedical Technology Co., Ltd. (Shanghai, China) for the proteomics analysis. Total protein of each sample was extracted, quantified, reduced, alkylated and digested to peptides. The peptides were labeled with an iodoTMTsixplex Isobaric Mass Tag Labeling Kit (Thermo Fisher Scientific, Waltham, MA, USA) according to the manufacturer’s instructions, and then desalted. A 2-µg sample of the TMT6-labeled peptides was transferred using a nano-ultra high-performance liquid chromatography (UHPLC) coupled to a Q Exactive HFX Orbitrap instrument (Thermo Fisher Scientific) equipped with a nano-electrospray ion source. The obtained mass spectrometry data were introduced into Proteome Discoverer software (PD) (version 1.4.0.288; Thermo Fisher Scientific) for screening. The screening parameters were set as follows: parent ion mass range: 400–5000 Da; minimum number of peaks in the secondary mass spectrum: 10; signal-to-noise ratio (S/N) threshold: 1.5. The mass spectrum obtained from the PD screening was further searched using the Mascot software search engine (version 2.3.2), followed by qualitative and quantitative analyses based on the UniProt database [12]. The original data included 5892 proteins extracted from six experimental samples (Additional file 1: Table S1), of which 5875 proteins were retained after pretreatment (Additional file 2: Table S2). DEPs were screened according to the following criteria: *P* value < 0.05 and foldchange < 0.83 or > 1.2, as described in other studies [13–16] (Additional file 3: Table S3). The DEPs were subjected to GO enrichment [17] and KEGG pathway enrichment analyses [18].

### Nontargeted metabolome analysis

Ant-1174 and Ant-Ct were injected into the female adult mosquitoes as described above for the proteomic analysis. Female mosquitoes were also collected before a blood meal at 72 h PIJ. Both the Ant-1174-injected group (Ant-1174) and control group contained eight biological replicates with 30 female mosquitoes in each group, yielding 16 Eppendorf tubes of samples prepared for liquid chromatography–tandem mass spectrometry (LC–MS/MS) nontarget metabolomics. All collected samples were frozen in liquid nitrogen, wrapped in dry ice and sent to the Shanghai Biotree Biotechnology Co., Ltd. for the LC–MS/MS analyses. To extract the metabolites, 50 mg of each sample were weighed into one Eppendorf tube containing 1000 μl of extract solution (acetonitrile:methanol:water = 2:2:1, with an isotope-labeled internal standard mixture). A mixture of an equal aliquot of the supernatants from all of the samples served as the quality control (QC) sample. All of the samples were analyzed on an UHPLC Vanquish system (Thermo Fisher Scientific) with a UPLC BEH Amide column (2.1 × 100 mm, 1.7 μm) coupled to Q Exactive HFX mass spectrometer (Thermo Fisher Scientific). We adopted both the positive ion mode (POS) and negative ion mode (NEG) in the QE ionization source of the electrospray ionization (ESI) system. Mobile phase A consisted of 25 mM/l ammonium acetate or 25 mM/l ammonia hydroxide in water (pH = 9.75) for the POS and NEG modes, respectively. Mobile phase B was acetonitrile for both modes. The elution gradient for the analysis was set as follows: 0–0.5 min, 95% B; 0.5–7.0 min, 95–65% B; 7.0–8.0 min, 65–40% B; 8.0–9.0 min, 40% B; 9.0–9.1 min, 40–95% B; 9.1–12.0 min, 95% B. The column temperature was 30 °C; the auto-sampler temperature was 4 °C; and the injection volume was 2 μl. A Q Exactive HFX mass spectrometer was used to evaluate MS/MS spectra in the information-dependent acquisition mode under control of the acquisition software Xcalibur (Thermo Fisher Scientific). The ESI source conditions were set as follows: sheath gas flow rate: 50 Arb; Aux gas flow rate: 10 Arb; capillary temperature: 320 °C; full MS resolution: 60,000; MS/MS resolution: 7500; collision energy: 10/30/60 in NCE mode; spray voltage: 3.5 kV (positive) or − 3.2 kV (negative).

The metabolomics raw data described above were converted to the mzXML format using ProteoWizard software [19] and then processed with the R package XCMS (v3.5) XCMS program [19], followed by peak pretreatments including detection, extraction, alignment and integration. The remaining peaks were annotated by comparison to the retention time and mass-to-charge ratio (*m*/*z*) indexes in the online databases HMDB (https://hmdb.ca/) [20] and KEGG (http://www.genome.jp/kegg) as well as in the in-house MS2 database (Biotree DB). The cut-off for annotation was set at 0.3. In the POS mode, 7003 peaks were extracted from three QC samples and 16 experimental samples, and 5714 peaks were retained after pretreatment (Additional file 4: Table S4). In the NEG mode, 7179 peaks were extracted from three QC samples and 16 experimental samples, and 5609 peaks were retained after pretreatment (Additional file 5: Table S5). In total, 14,113 peaks were extracted from the combined POS and NEG modes, and 11,270 peaks were retained after pretreatment (Additional file 6: Table S6). The data from the POS and NEG modes were finally merged in the subsequent data analysis.

Principle component analysis (PCA) [21], an unsupervised analysis which reduces the dimensions of the data, was carried out to visualize the distribution and the grouping of the samples. A 95% confidence interval (CI) (Hotelling’s T-squared ellipse) in the PCA score plot was used as the threshold to identify potential outliers in the dataset. Orthogonal Partial Least Squares-Discriminant Analysis (OPLS-DA) [22, 23] was applied to further visualize the group separation and identify significantly changed metabolites. The metabolites with variable importance in projection (VIP) > 1 and *P* < 0.05 (unpaired two-sided Student’s t-tests) in the OPLS-DA model were considered to be differentially expressed metabolites (DEMs). Volcano plots and a hierarchically clustered heatmap were plotted to visualize significant features of the identified DEMs. Commercial databases, including KEGG (http://www.genome.jp/kegg/) and MetaboAnalyst 5.0 (http://www.metaboanalyst.ca/), were used for pathway enrichment analysis. Finally, integrated analysis of the proteome and metabolome was performed for the differential proteins and metabolites.

### Target prediction and confirmation

We downloaded the 3′-untranslated regions (3’-UTRs) of *Ae. aegypti* from VectorBase (https://vectorbase.org/vectorbase/app) and predicted the candidate targets of miR-1174 using RNAhybrid [24], miRanda [25], TargetScan [26] and Target Accessibility (PITA) [27]. The predicted candidate targets were crossed with the upregulated proteins, yielding the final candidate targets. The 3′-UTRs of these candidate target genes were amplified by rapid amplification of cDNA ends (3′-RACEs), and cloned into the* psiCHECK™-2* vector after digestion with the restriction enzymes *Not *I and *Xho *I. The miR-1174 mimic used for cell transfection was purchased from Thermo Fisher Dharmacon (Thermo Fisher Scientific). The three candidate target genes were finally verified by dual-luciferase reporter assay on GloMax®-Multi Detection System (Promega, Madison, WI, USA), as described in our previous work [28]. The cell line HEK-293T, vector *psiCHECK™-2*, and reagents for the dual-luciferase reporter assay (Dual-Glo^®^ Luciferase Assay Reagent and Dual-Glo^®^ Stop & Glo^®^ Reagent) were purchased from Promega.

### RNA extraction and real time quantitative PCR

Total RNA was extracted using TRIzol Reagent (Life Technologies, Carisbad, CA, USA) following the manufacturer’s manual and then quantified using a Nano 300 Spectrophotometer (Thermo Fisher Scientific). RNA with an optical density (OD) 260/280 > 1.8 was considered to be satisfactory for subsequent experiments. The integrity of the extracted RNA was checked by 1% agarose gel electrophoresis. All primer pairs for the real time quantitative PCR (RT-qPCR) amplification of genes were designed using the software Primer Premier 5.0 and then synthesized at Sangon Biotech (Shanghai, China) (Additional file 7: Table S7). To examine the expression of the protein-coding genes, 1 µg of total RNA was reverse-transcribed into complementary DNA (cDNA) using the SuperScript™ IV First-Strand Synthesis System (TaKaRa Bio Inc., Otsu, Shiga, Japan). RT-qPCR was performed using TB Green Premix Ex Taq TM II (Tli RNaseH Plus; TaKaRa Bio Inc.). The reaction volume (20 µl) contained 10 µl of 2× NovoStart® SYBR qPCR SuperMix Plus (Tiangen Biotechnology Co., Ltd., Beijing, China), 0.8 µl of 10 µM primers, 0.4 µl ROX Dye II, 2 µl diluted cDNA and 6.8 µl ddH_2_O. Ribosomal protein S7 (RPS7; AAEL009496-RA) served as the internal control. Reactions were conducted on an ABI7500 real-time PCR system (Applied Biosystems, Thermo Fisher Scientific). The amplification procedure was: pretreatment at 95 °C for 1 min; followed by 40 cycles of 95 °C for 1 min and 60 °C for 1 min; ending with the generation of the dissolution curves at 95 °C for 15 s, 60 °C for 1 min and 95 °C for 15 s. To examine the expression level of miR-1174, 1 µg of total RNA was reverse-transcribed into cDNA using the SynScript^®^ III miRNA RT SuperMix (TSK3001; Tsingke Biotechnology Co.,Ltd., Beijing, China). RT-qPCR was performed using a miRNA Universal SYBR qPCR Mix (TSE2001; Tsingke Biotechnology Co., Ltd.). The total reaction volume (20 µl) contained 10 µl of miRNA Universal SYBR qPCR Mix (Tsingke Biotechnology Co., Ltd.), 0.8 µl of 10 µM primers, 0.4 µl of 50× ROX Reference Dye II, 2 µl of diluted cDNA and 6.8 µl of ddH_2_O. Small nuclear RNA (snRNA) U6 (AAEL029000-RA) was used as the internal control. Reactions were conducted on the ABI7500 real-time PCR system (Applied Biosystems, Thermo Fisher Scientific) using the following amplification procedure: pretreatment at 95 °C for 1 min; followed by 40 cycles of 95 °C for 10 s and 60 °C for 30 s; ending with the generation of the dissolution curves at 95 °C for 15 s, 60 °C for 1 min and 95 °C for 15 s.

### Preparation and injection of the double-stranded RNA

Total RNA was extracted from mosquitoes with TRIzol reagent as described above. The first-strand cDNA was generated using SuperScript™ IV First-Strand Synthesis System (TaKaRa Bio Inc.). PCR was performed with Platinum™ PCR SuperMix High Fidelity mixture (Invitrogen, Thermo Fisher Scientific) according to the following procedures: 94 °C for 2 min, 40 cycles of 94 °C for 30 s, 55 °C for 30 s and 68 °C for 1 min; and a final extension at 72 °C for 7 min. The double-stranded RNA (dsRNA) was synthesized with a MEGAScript RNAi Kit (Thermo Fisher Scientific) and then dissolved in RNase-free water to a concentration of approximately 1800 ng/μl. dsRNA quality was determined by 1% agarose gel electrophoresis, and the dsRNA meeting the standards was stored at - 80 °C before use. Each female mosquito was injected with 0.5 μl of dsRNA at about 16 h PE. dsRNA of enhanced green fluorescent protein (ds*EGFP*) at similar concentrations as the test dsRNA served as a control. Three days after injection, females were collected for total RNA extraction and RT-qPCR assay. Three biological replicates each were used for the treatment and control groups, with five female mosquitoes in one tube.

### Prokaryotic expression of *PNP* for its antibody preparation

The expression vector *pET-28a(+) *was stored in our laboratory [8], and the clone strain Trans-T1 and expression strain BL21 were purchased from TransGen Biotech Co., Ltd. (Beijing, China). Total RNA of adult female mosquitoes at 72 h PIJ was used for the cDNA synthesis. Primers with the restriction sites *Not *I and *Nco *I for *PNP* coding sequence (CDS) cloning were designed using Primer Premier 5.0 software (Additional file 7: Table S7). PCR was conducted using PrimeStar HS DNA Polymerase (Takara Bio Inc.) as follows: 94 °C for 3 min; followed by 25 cycles of 94 °C 30 s, 65 °C 30 s and 72 °C 3 min; and 72 °C for 10 min. After verification by double-enzyme digestion, the recombinant plasmid was transformed into expressing strain BL21 to facilitate the prokaryotic expression of *PNP*. 0.1% IPTG was added for induction, and the mixture was incubated at 37 °C for 4 h. The protein was purified based on affinity chromatography on a Ni^2+^ ion-bound chromatography column (General Electric Company, Boston, MA, USA) and eluted with imidazole at different concentrations (200 mM, 500 mM, 1 M, 2 M). The purified protein was dialyzed to remove the imidazole and was finally sent to Wuhan GeneCreate Biological Engineering Co., Ltd. (Wuhan, China) for the preparation of antibody.

### Protein extraction and western blotting

Mosquitoes were ground thoroughly in RIPA lysis buffer (50 mM Tris–HCl, pH 7.5, 150 mM NaCl, 2 mM EDTA, 0.1% sodium dodecyl sulfate [SDS], 1% Triton X-100, 0.5% sodium deoxycholate and EDTA-free protease inhibitor；Beyotime Biotechnology Co., Ltd., Shanghai, China) using an electric homogenizer grinder (TGrinder OSE-Y40; Tiangen Biotechnology Co., Ltd.). Protein concentrations were assessed with the BCA Assay Kit (Beyotime Biotechnology Co., Ltd.) on a microplate reader (Thermo Fisher Scientific). For each sample, 40 ng of total protein (lysate) was mixed with one-fifth volume of 5× SDS loading buffer and then boiled in a waterbath at 100 °C for 10 min, followed by separation in 12% SDS-polyacrylamide electrophoresis (PAGE) gels, transfer of the electrophoresis products onto a polyvinylidene difluoride (PVDF) membrane and then blocking of the membrane with 5% skimmed milk at room temperature for 2 h. The primary antibody diluted with 1× Tris buffered saline with Tween 20 buffer (TBST, pH7.4) at 8000:1 was added to 1% nonfat milk powder prepared with 1× TBST and incubated at 4 °C overnight or at room temperature for 1 h. Blots were washed 3 times with 1× TBST for 10 min each, and then incubated with 0.2 μg/ml anti-rabbit antibody diluted with TBST at 10,000:1 for 1 h at room temperature. Blots were then washed 6 times with 1× TBST for 10 min each. Specific bands were developed with a Clarity Western ECL Substrate and detected using ChemiDoc XRS (Bio-Rad Laboratories, Hercules, CA, USA). Densitometry analysis of protein bands corresponding to the *PNP* were quantified with Image J Software (National Institutes of Health, Bethesda, MD, USA). Tubulin (AT819; Beyotime Biotechnology Co., Ltd.) served as a loading control in western blot experiments.

### Measurement of triglyceride content

Triglyceride contents (TGs) were quantified using the TG Colourimetric Assay Kit (Cat. No.: D799796-0100; Sangon Biotech, Shanghai, China) in accordance with the manufacturer’s instructions. Briefly, the fat bodies of eight female mosquitoes at 36 h post blood meal (PBM) were harvested as one biological replicate for ds*PNP* or ds*EGFP*, and were homogenized thoroughly in 240 µl of lysis buffer (mixture of* n*-heptane and isopropanol in a 1:1 volume ratio) with an electric homogenizer (TGrinder OSE-Y40; Tiangen Biotech Co., Ltd.), followed by centrifugation at 12,000 *g* for 15 min to remove the fat layer. A 120-µl sample of the supernatant was transferred to a new 1.5-ml Eppendorf tube and sequentially treated with reagents 1–6 provided in the TG Colourimetric Assay Kit before measuring the absorbance value at 420 nm on the assay instrument (GloMax®-Multi Instrument YNERGY H4; Promega).

### Measurement of ATP levels

ATP levels were measured using the ATP Assay Kit (Cat. No.: S0026; Beyotime Biotechnology Co., Ltd.) according to the manufacturer’s instructions. Briefly, the fat bodies of eight female mosquitoes at 36 h PBM were pooled into a 1.5-ml Eppendorf tube as one biological replicate, followed by grinding in 120 μl of lysate with an electric homogenizer (TGrinder OSE-Y40; Tiangen Biotech Co., Ltd.). After centrifugation at 16,000 *g* for 5 min at 4 °C, the supernatant was transferred to a new 1.5-ml Eppendorf tube and 100 μl of the diluted ATP detection reagent was added to each well in a 96-well plate. The plate was maintained at room temperature for 3–5 min so that the background ATP was consumed in the reaction. Then, 20 μl of the supernatant sample or standard sample was added to each well, quickly mixed and maintained at room temperature for at least 2 s, following which the relative light unit (RLU) was measured by an ELISA reader (GloMax^®^-Multi Instrument; Promega). The ATP content in each sample was calculated on the standard curve, which was generated by the following concentration gradients of ATP standard solution: 0.1 μM, 0.15 μM, 0.3 μM, 0.65 μM, 1.5 μM and 3 μM. All values were normalized to the control group.

### Statistics and reproducibility

All statistical analyses were performed using GraphPad Prism 7.0 (GraphPad Software, La Jolla, CA, USA) and the values presented as the mean ± standard error of the mean (SEM). The experiments were repeated at least three times, and each experiment included at least three treatments. The error bars were generated from experimental replicates. The mean cycle threshold (Ct) value was converted to relative expression level using the 2^−ΔΔCt^ method [29, 30]. Statistical analyses of the expression levels were performed using a two-tailed unpaired Student t-test. Significant differences were defined as *P < 0.05, **P < 0.01 and ***P < 0.001.

## Results

### Differentially expressed proteins in miR-1174-depleted mosquitoes

**Fig. 1:**
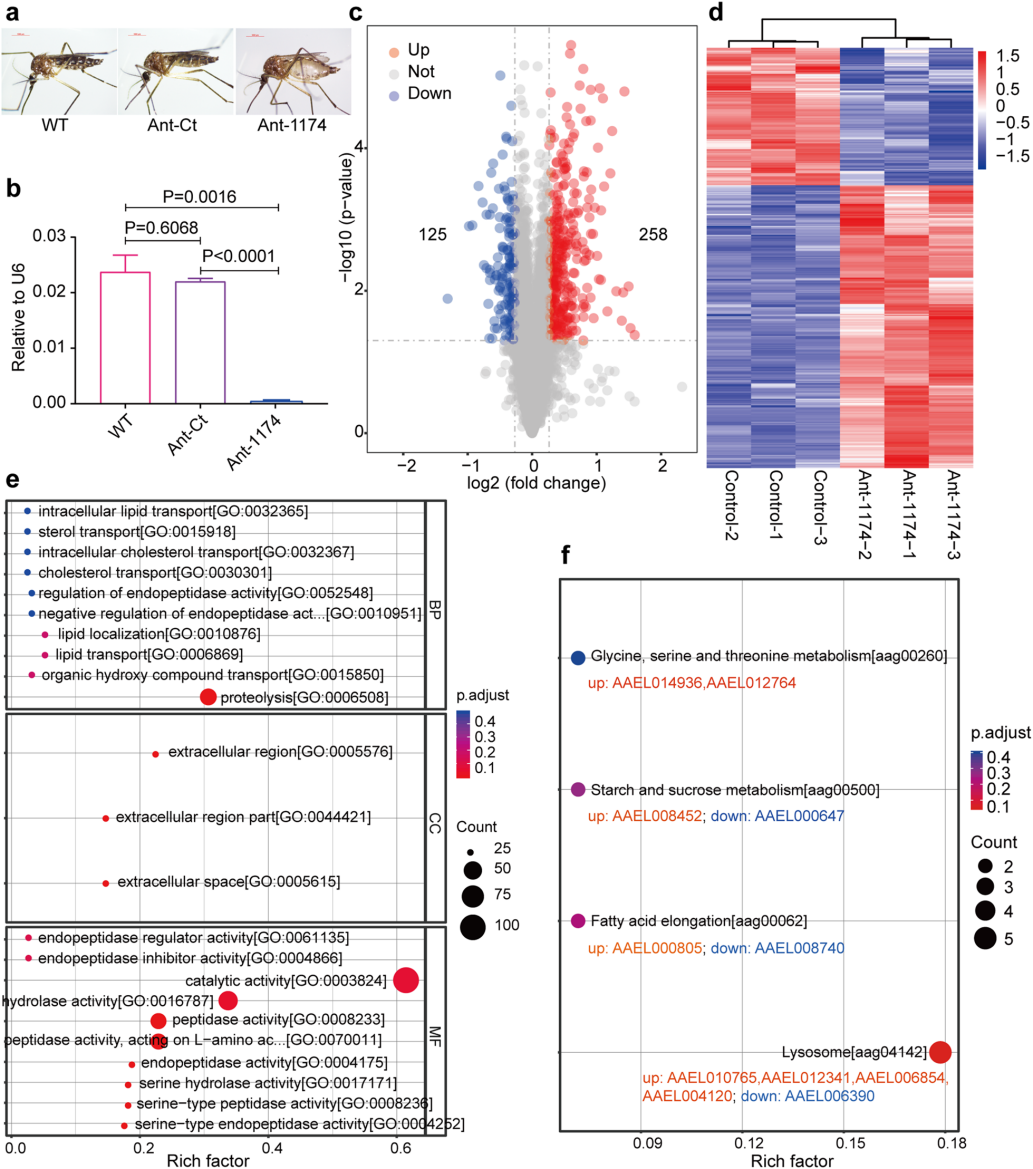
Screening of differentially expressed proteins after miR-1174 depletion. **a** Ant-1174-injected mosquitoes and controls at 72 h PIJ. **b** miR-1174 levels in the Ant-1174-injected mosquitoes and controls. Data are shown as the mean ± SEM of 3 independent experiments. **c** Volcano plot (fold change > 1.2 or < 0.83, *P* < 0.05) illustrating the proteins differentially expressed between the Ant-1174 and control groups. The significantly upregulated differentially expressed proteins are shown in red, the significantly downregulated differentially expressed proteins are shown in blue and the proteins with no significant difference are shown in gray. **d** Hierarchical clustering analysis of the differentially expressed proteins visualized as a heatmap. The color blocks in different positions represent the relative expression of the corresponding proteins: red represents high expression and blue represents low expression. **e** Bubble chart showing GO enrichment of Ant-1174 vs Control. **f** KEGG pathways enriched by differentially expressed proteins between Ant-1174 and control group. Ant-1174/Ant-Ct, antisense oligonucleotides (antagomirs) Ant-1174/Ant-Ct; BP, biological Process; CC, cellular component; GO, Gene Ontology; KEGG, Kyoto Encyclopedia of Genes and Genomes; MF, molecular function; miRNA-1174, microRNA-1174; PIJ, postinjection; WT, wild type

We collected the Ant-1174-injected and control mosquitoes before a blood meal at 72 h PIJ (Fig. 1a) for the RT-qPCR assay of miR-1174 expression. Both our previous studies [7] and the present experiment showed that the expression level miR-1174 was significantly reduced by the injection of Ant-1174 (Fig. 1b). Three days after the treatment, the injection wound had fully recovered, and there were no differences in the mosquito phenotypes ormiR-1174 expression between the Ant-Ct-injected group and the non-injected wild-type group (Fig. 1a, b) [7]. Three biological replicates of Ant-1174–injected and Ant-Ct control mosquitoes were collected for the proteomic analysis. A total of 5892 proteins were detected in these six samples, of which 5875 proteins remained after data filtering and normalization. Using differential protein expression analysis with the criteria of *P* value < 0.05 and fold-change < 0.83 or > 1.2, as used in other studies [13–16], we determined 383 DEPs, of which 258 were upregulated and 125 were downregulated (Fig. 1c; Additional file 8: Table S8). Hierarchical cluster analysis showed that these 383 DEPs were separated into upregulated and downregulated clades, and that the Ant-1174 group was differentiated from the control group (Fig. 1d); thus, miR-1174 depletion substantially caused a global protein expression response in mosquitoes.

We performed functional enrichment analysis of these 383 DEPs by GO and found that 137, 166 and 88 DEPs were enriched in biological processes, cellular components and molecular function, respectively (Fig. 1e). Functional analysis of the DEPs showed that the catalytic activity pathway was the most enriched biological process among the DEPs, followed by the hydrolase activity pathway. Serine-related pathways serine hydrolase activity, serine-type peptidase and serine-type endopeptidase activity were also enriched. Functional analysis also suggested that miR-1174 depletion affected amino acid metabolism and the catalytic response of other enzymes. KEGG pathway enrichment analysis showed that five proteins were enriched in the lysosome pathway (aag04142), two proteins were enriched in the fatty acid elongation pathway (aag00062) and two proteins were enriched in the starch and sucrose metabolism pathway (aag00500) (Fig. 1f). In the enriched lysosome pathway, four proteins were upregulated (AAEL010765, AAEL012341, AAEL006854 and AAEL004120) and one was downregulated (AAEL006390) (Fig. 1f). In summary, the results of both GO enrichment analysis and KEGG Pathway enrichment analysis suggested that miR-1174 plays important regulatory roles in the amino acid metabolism, nucleotide metabolism, fatty acid metabolism and sugar metabolism pathways.

### Differential metabolites identified in miR-1174-depleted mosquitoes

**Fig. 2:**
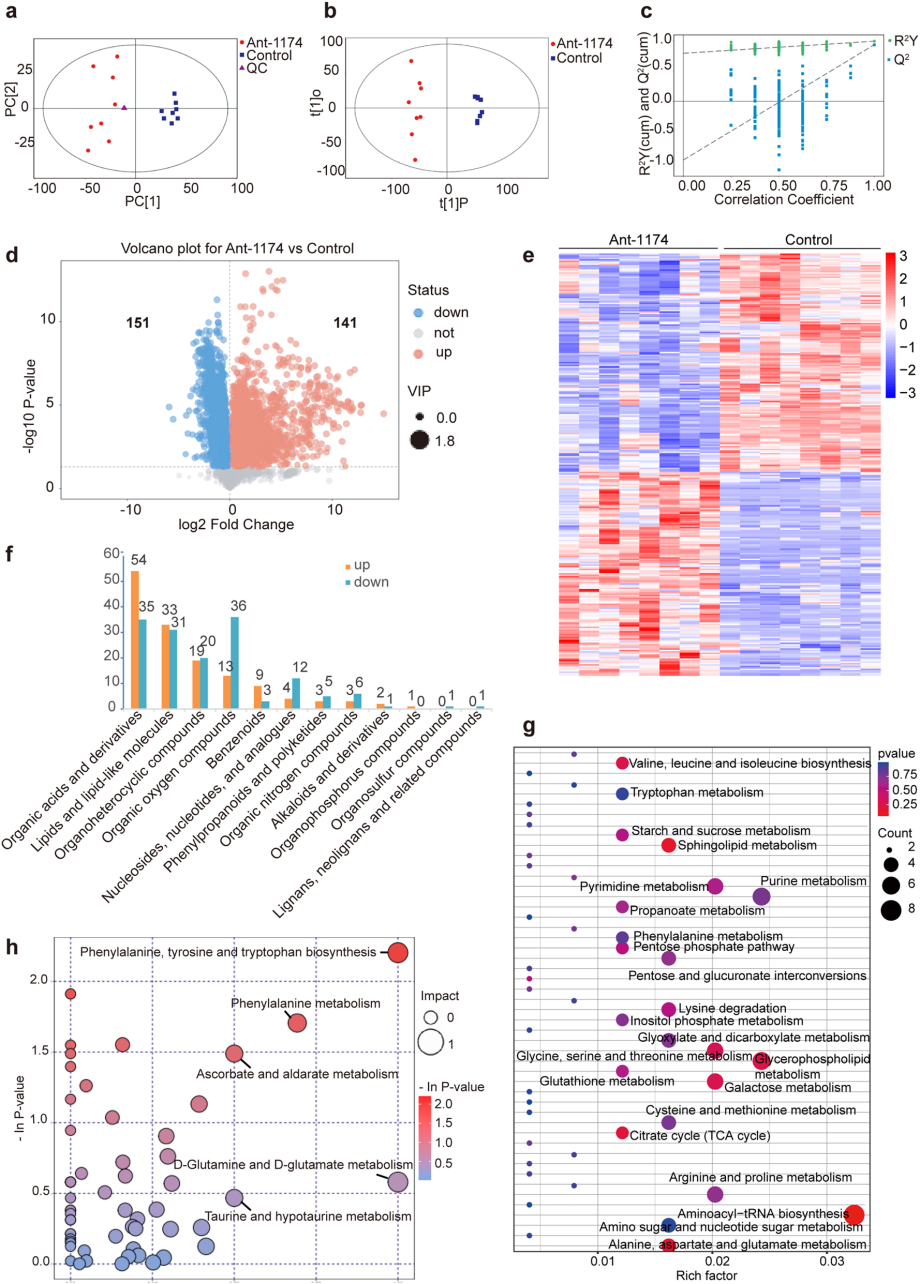
Metabolomic profiling of miR-1174-depleted mosquitoes. **a** Score scatter plot of PCA model for groups with quality control. All samples were within the 95% confidence interval (Hotelling's T-squared ellipse).** b** Score scatter plot of OPLS-DA model for Ant-1174 vs Control. **c** Permutation test of OPLS-DA model between Ant-1174 and control groups. Intercepts: *R*^2^*Y*(cum) = (0, 0.79), *Q*^2^(cum) = (0, − 0.97). **d** Volcano plot of differential metabolites. Red dots represent upregulated metabolites, blue dots are downregulated ones. The *X*-axis indicates log_2_ fold change, and the *Y*-axis measures the − log_10_ *P*-value. **e** Heatmap of differential metabolites. Downregulated metabolites are highlighted in blue and clustered in the upper left, upregulated metabolites are represented in red and clustered in the lower left. **f** Classification of upregulated metabolites and downregulated metabolites. **g** KEGG pathways enriched by the differential metabolites. The circle size is directly proportional to the number of differential metabolites; the depth of red is inversely proportional to the* P*-value. **h** Metabolic pathway analysis of differential metabolites. In the bubble diagram, each bubble represents a metabolic pathway, and the size of the bubble represents the influence factor size of the pathway in topological analysis. Ant-1174, Antisense oligonucleotide (antagomirs) Ant-1174; KEGG, Kyoto Encyclopedia of Genes and Genomes; miR-1174, microRNA-1174; OPLS-DA, orthogonal projections to latent structures- discriminant analysis; PCA, principal component analysis

Next, we performed untargeted metabolomics analysis of the Ant-1174 and control groups to elucidate the differential metabolites which resuled from miR-1174 depletion in *Ae. aegypti* mosquitoes. All samples in the PCA score plot were within the 95% CI (Fig. 2a), confirming that the PCA model for our data was statistically reliable. Similarly, according to the OPLS-DA diagram, all the samples were within the 95% CI with *R*^2^*Y* = 0.988 and *Q*^2^ = 0.931 (Fig. 2b), confirming that the OPLS-DA model to identify the differential metabolites was reliable. Moreover, on the replacement test diagram, the *R*^2^*Y* and *Q*^2^ values of the original OPLS-DA model were greater than the *R*^2^*Y* and *Q*^2^ values of the replacement test diagram, and the intercept of the *Q*^2^ regression line and the vertical axis was less than zero (Fig. 2c). These observations indicate that the established OPLS-DA model was not overfit and had good predictability.

Differential screening by VIP (in an OPLS-DA model) > 1 and *P* value (in a Student’s t-test) < 0.05 yielded 292 differential metabolites, of which 141 were upregulated and 151 were downregulated (Fig. 2d; Additional file 9: Table S9). Hierarchical clustering analysis of these differential metabolites showed that samples of the same group were clustered in one clade and those of different groups were separated from each other (Fig. 2e; Additional file 10: Table S10). The upregulated metabolites could be divided into 10 super classes, of which the top five were organic acids and their derivatives; lipids and lipid-like molecules; organoheterocyclic compounds; organic oxygen compounds; and benzenoids (Fig. 2f; Additional file 11: Table S11). The downregulated metabolites could be divided into 11 super classes, of which the top five were organic oxygen compounds; organic acids and derivatives; lipids and lipid-like molecules; organoheterocyclic compounds; and nucleosides, nucleotides and analogs (Fig. 2f; Additional file 11: Table S11). In the super class organic acids and derivatives, 41 metabolites (76%) of POS mode and 24 metabolites (69%) of NEG mode belonged to the subclass amino acids, peptides and analogs. In the super class organic oxygen compounds, eight metabolites (62%) of POS mode and 29 metabolites (81%) of NEG mode belonged to the subclass carbohydrates and carbohydrate conjugates. We then performed a KEGG pathway enrichment analysis of these DEMs (Fig. 2g; Additional file 12: Table S12), which revealed that the DEMs were mainly enriched in the pathway aminoacyl-tRNA biosynthesis, followed by purine metabolism, glycerophospholipid metabolism, galactose metabolism, pyrimidine metabolism, glycine, serine and threonine metabolism, arginine and proline metabolism, pentose and glucuronate interconversions, alanine, aspartate and glutamate metabolism, cysteine and methionine metabolism, lysine degradation, amino sugar and nucleotide sugar metabolism, sphingolipid metabolism and glyoxylate and dicarboxylate metabolism.

In the purine metabolism pathway, urate and hypoxanthine ribonucleotides were upregulated, and glyoxylate, d-ribose 5-phosphate, adenine nucleoside diphosphate, adenosine 3′,5′-diphosphate, adenosine, aminimidazole riosse and 3′-adenosine phosphate were downregulated. Uracil ribonucleotides, cytosine ribonucleotides and cytidine in the pyrimidine metabolism pathway were also downregulated. In the glycine, serine and threonine metabolism pathway, l-threonine and aminoacetone were upregulated, while phosphate-l-serine, phosphate-l-hyperserine and l-serine were downregulated. Serine is an important precursor for the synthesis of purines, pyrimidines and phospholipids, and downregulation of serine and its derivatives could affect DNA replication. Further analysis showed that the pathways most closely related to miR-1174 downregulation were the phenylalanine, tyrosine and tryptophan biosynthesis pathway and the phenylalanine metabolism pathway. In these pathways, l-phenylalanine and phenylacetaldehyde were upregulated, and l-tyrosine and d-erythrose 4-phosphate were downregulated (Fig. 2h).

Downregulation of miR-1174 also changed the accumulation of compounds in the purine metabolism (aag00230) and pyrimidine metabolism (aag00240) pathways. In the purine metabolism pathway, d-ribose 5-phosphate, ADP, adenosine, adenosine 3',5'-diphosphate, aminoimidazole ribonucleotide and 3'-AMP were downregulated. In the pyrimidine pathway, 4,5-dihydroorotic acid was upregulated and uridine 5'-monophosphate, cytidine monophosphate, cytidine and beta-alanine were downregulated. We therefore speculate that the abnormal phenotypes observed in miR-1174-depleted mosquitoes might be caused by a widespread disorder involving both amino acids and nucleotides.

### Integrative analysis of differential proteins and differential metabolites

**Fig. 3:**
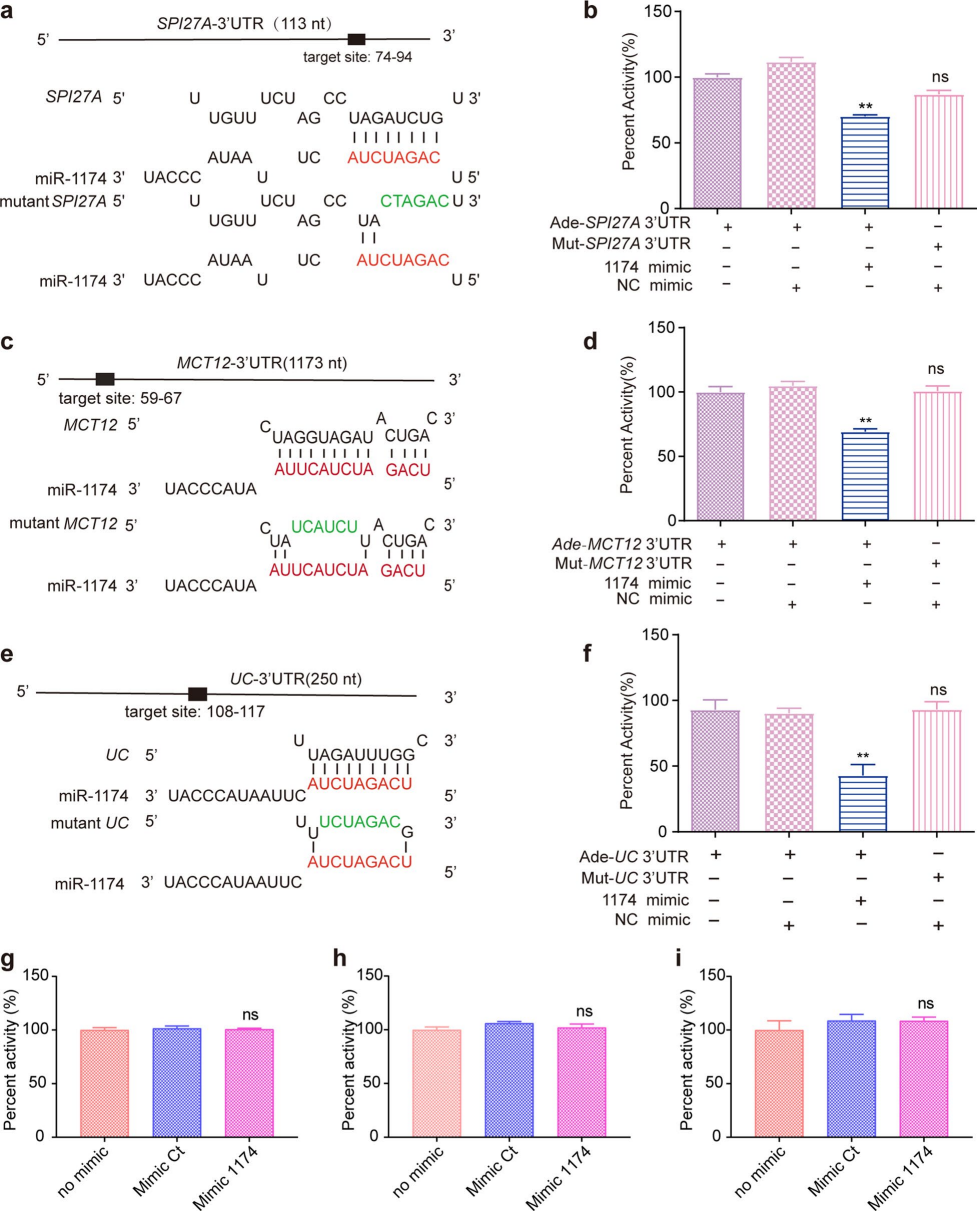
Target genes of miR-1174. **a** Predicted binding site within the 3’-UTR of serine protease inhibitor 27A (*SPI27A*, AAEL014079). **b** Dual-luciferase reporter assay of the binding site within the 3’-UTR of *SPI27A* mRNA. **c** Predicted binding site within the 3′-UTR of *MCT12* mRNA. **d** Dual-luciferase reporter assay of the binding site within the 3′UTR of *MCT12* mRNA. **e** Predicted binding site within the 3’-UTR of uncharacteristic (*UC*, AAEL015019). **f** Dual-luciferase reporter assay of the binding site within the 3′UTR of *UC* mRNA. **g** Dual-luciferase reporter assay to check whether miR-1174 targets the 5’-UTR of *PNP* mRNA. **h** Dual-luciferase reporter assay to check whether miR-1174 targets the CDS of *PNP* mRNA. **i** Dual-luciferase reporter assay to check whether miR-1174 targets the 3’UTR of *PNP* mRNA. Error bars depict ± SEM of three independent experiments. Asterisks indicate statistical significance at *P < 0.05 and **P < 0.01; ns, not significant. CDS, Coding sequence; miR-1174, microRNA-1174; UTR, untranslated region; mRNA, messenger RNA; PNP, purine nucleoside phosphorylase; SEM, standard error of the mean; 3′-UTR, 3′-untranslated region

Given that many DEPs and DEMs were enriched in amino acid metabolism, purine metabolism and pyrimidine metabolism pathways, we focused on these three pathways for the integrative analysis of DEPs and DEMs (Fig. 3). Of note, in the purine metabolism pathway (aag00230), the associated proteins purine nucleoside phosphorylase (PNP, aag5574096) and GTP:AMP phosphotransferase AK3 (PAK3, aag5564849) were upregulated, along with the associated upregulated metabolites inosinic acid or inosine monophosphate (IMP, cpdC00130) and uric acid or urate (cpdC00366), whereas the associated downregulated metabolites were ADP (cpdC00008), glyoxylate (cpdC00048), adenosine 3',5'-bisphosphate (cpdC00054), d-ribose 5-phosphate (cpdC00117), adenosine (cpdC00212), 3'-AMP (cpdC01367) and aminoimidazole ribotide (AIR, cpdC03373). In the pyrimidine metabolism pathway (aag00240), the associated upregulated protein was PNP (aag5574096), and the associated upregulated metabolite was 4,5-dihydroorotic acid or (*S*)-dihydroorotate (cpdC00337). In the glycine, serine and threonine metabolism pathway (aag00260), the associated proteins glycine* N*-methyltransferase (GNMT, aag5576784) and sarcosine dehydrogenase (AARDH, aag5565677) were upregulated, and the associated metabolites l-threonine (cpdC00188), betaine (cpdC00719) and aminoacetone (cpd C01888) were upregulated, while the associated metabolites glyoxylic acid or glyoxylate (cpdC00048), l-serine (cpdC00065), 2-oxobutanoate (cpd C00109), d-glycerate (cpd C00258),* O*-phospho-l-serine (cpdC01005) and * O*-phospho-l-homoserine (cpdC01102) were downregulated. Together, these associated DEPs and DEMs have important roles in amino acid metabolism and DNA metabolism. Therefore, it is possible that miR-1174 depletion may exert a wide impact on the metabolic pathways of amino acids and nucleotides in mosquitoes, consequently affecting blood sucking, blood digestion and ovarian development.

### Three targets of miR-1174

The candidate targets predicted by using four programs (see Methods) were intersected with all of the upregulated proteins in the miR-1174-depleted mosquitoes, yielding three candidate targets: serine protease inhibitor 27A (*SPI27A*, AAEL014079), monocarboxylate transporter 12 (*MCT12*, AAEL002412) and uncharacteristic (*UC*, AAEL015019). The 3'-UTR sequences of *SPI27A*, *MCT12* and *UC* were obtained by 3'-RACE and each was cloned into the psiCHECKTM-2 vector. HEK-293T cells were co-transfected with the recombinant plasmid and a miR-1174 mimic. Firefly luciferase activity (Fluc) and renilla luciferase activity (Rluc) were detected at 48 h after transfection. The results of the dual-luciferase reporter gene assay showed that the Rluc/Fluc ratio was significantly lower than that of the control group, showing that *SPI27A*, *MCT12* and *UC* were target genes of miR-1174 (Fig. 3a–f). We further mutated the predicted target sites of miR-1174 within their 3’-UTRs, and the results of dual-luciferase reporter gene assay showed that luciferase activity recovered after site mutation, which indicated that the mutant site was the target of miR-1174 (Fig. 3a–f). These three targets were knocked down by injection of double-stranded RNAs (dsRNAs), but no abnormal phenotypes were observed in the mosquitoes (data not shown). Whether these three target genes have important functions in mosquitoes needs to be further confirmed through gene knockout or overexpression.

### Knockdown of *PNP* inhibited blood digestion and ovary development

**Fig. 4:**
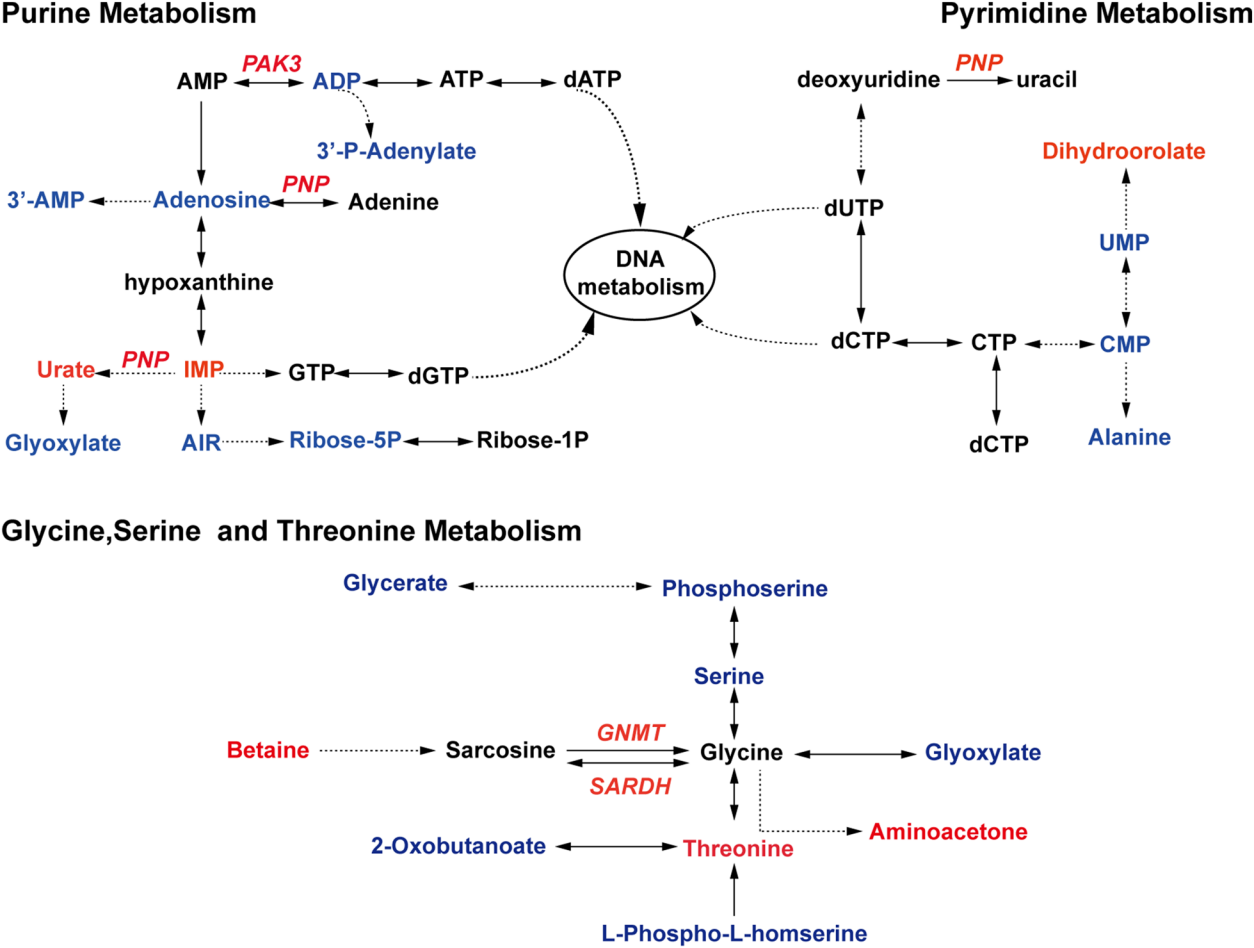
Integrated analysis of differentially expressed proteins and metabolites. Red indicates upregulation and blue indicates downregulation. The dotted line indicates multi-step reactions, and the solid line indicates one-step reactions. The two-way arrow represents the equivalence between different scenarios, while the one-way arrow means unidirectional transformation between substrate and product or from one metabolite to another. AIR, Aminoimidazole ribotide; IMP, inosine monophosphate; PAK3, GTP:AMP phosphotransferase; PNP, purine nucleoside phosphorylase; GNMT, glycine N-methyltransferase; SARDH, sarcosine dehydrogenase

**Fig. 5:**
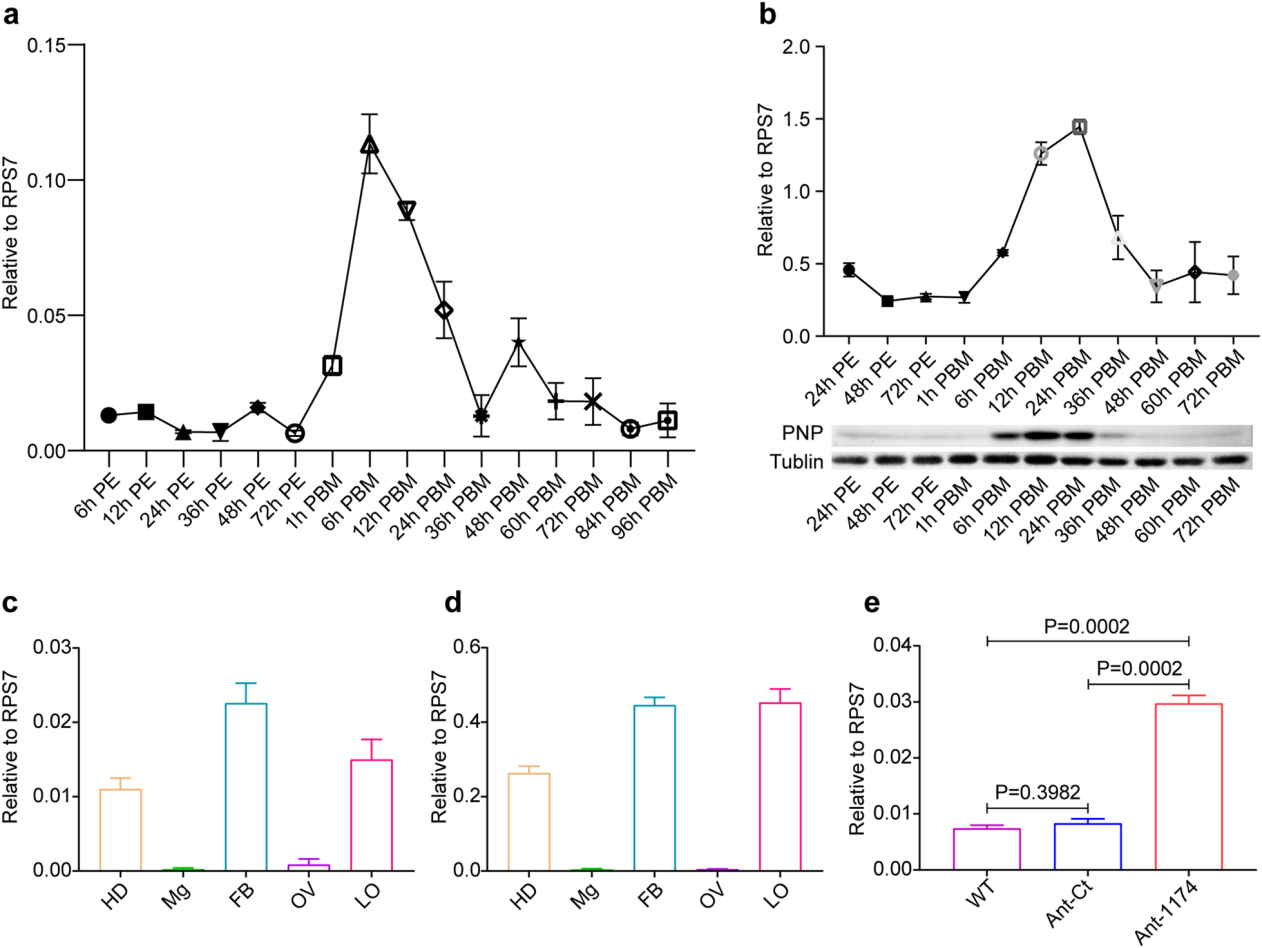
Expression of *PNP*. **a** RT-qPCR-based temporal expression of *PNP* in adult female mosquitoes. **b** Western blotting analysis of *PNP* in adult mosquitoes. In order to compare with the mRNA levels in **a**, the signals of the western blotting results (below) were converted into numbers using Image J software to quantify protein expression (above). **c** RT**-**qPCR-based spatial expression at 72 h PE. **d** RT-qPCR-based spatial expression at 6 h PBM. **e** Expression of *PNP* in response to miR-1174 depletion. Females were dissected in phosphate-buffered saline for tissue collection, and the blood boluseswere removed from the midgut. RPS7 was used as the control for RT-qPCR, and tubulin was used as the control for western blotting. All graphs are presented as mean ± SEM of 3 independent experiments. Differences at *P* < 0.05 were statistically significant, and differences at *P* < 0.01 were considered to be highly statistically significant. FB, Fat body; HD, head; LO, leftover; Mg: midgut; miR-1174,, microRNA-1174; PBM, post blood meal; PE, posteclosion; PNP, purine nucleoside phosphorylase; RT-qPCR, real-time quantitative PCR; SEM, standard error of the mean

Integrative analysis of the DEPs and DEMs showed that *PNP* (AAEL002269) was associated with the pathways for purine metabolism, pyrimidine metabolism and niacin-nicotinamide metabolism (Fig. 4), indicating that *PNP* might play important roles in mosquitoes. Therefore, we investigated spatiotemporal expression patterns of the *PNP* gene by RT-qPCR and western blotting assays. Female adult mosquitoes were collected at 14 time points for RT-qPCR assays; the results showed that the expression of *PNP* increased rapidly after mosquitoes took a blood meal, with the highest expression level at 6 h PBM and the lowest expression level at 48 h PBM (Fig. 5a). The protein level of* PNP* was robustly elevated, reaching a plateau from 12 to 24 h PBM and then decreasing to a trough at 48 h PBM; this trend was somewhat delayed compared with the mRNA level (Fig. 5b). In order to determine the spatial expression of *PNP*, we collected different tissues at 72 h PE and 6 h PBM for RT-qPCR and western blotting assays. The results showed that *PNP* was expressed at high levels in the fat body and head and displayed low expression levels in the midgut and ovary (Fig. 5c, d). *PNP* was significantly induced in response to the depletion of miR-1174 (Fig. 5e), implying that miR-1174 might negatively regulate *PNP*. We then predicted the binding interaction between miR-1174 and *PNP* but found no target sites within the 5’-UTR, CDS or 3’-UTR of *PNP* mRNA, a result which was further confirmed by the results of dual-luciferase reporter gene assay (Fig. 3g–i).

**Fig. 6:**
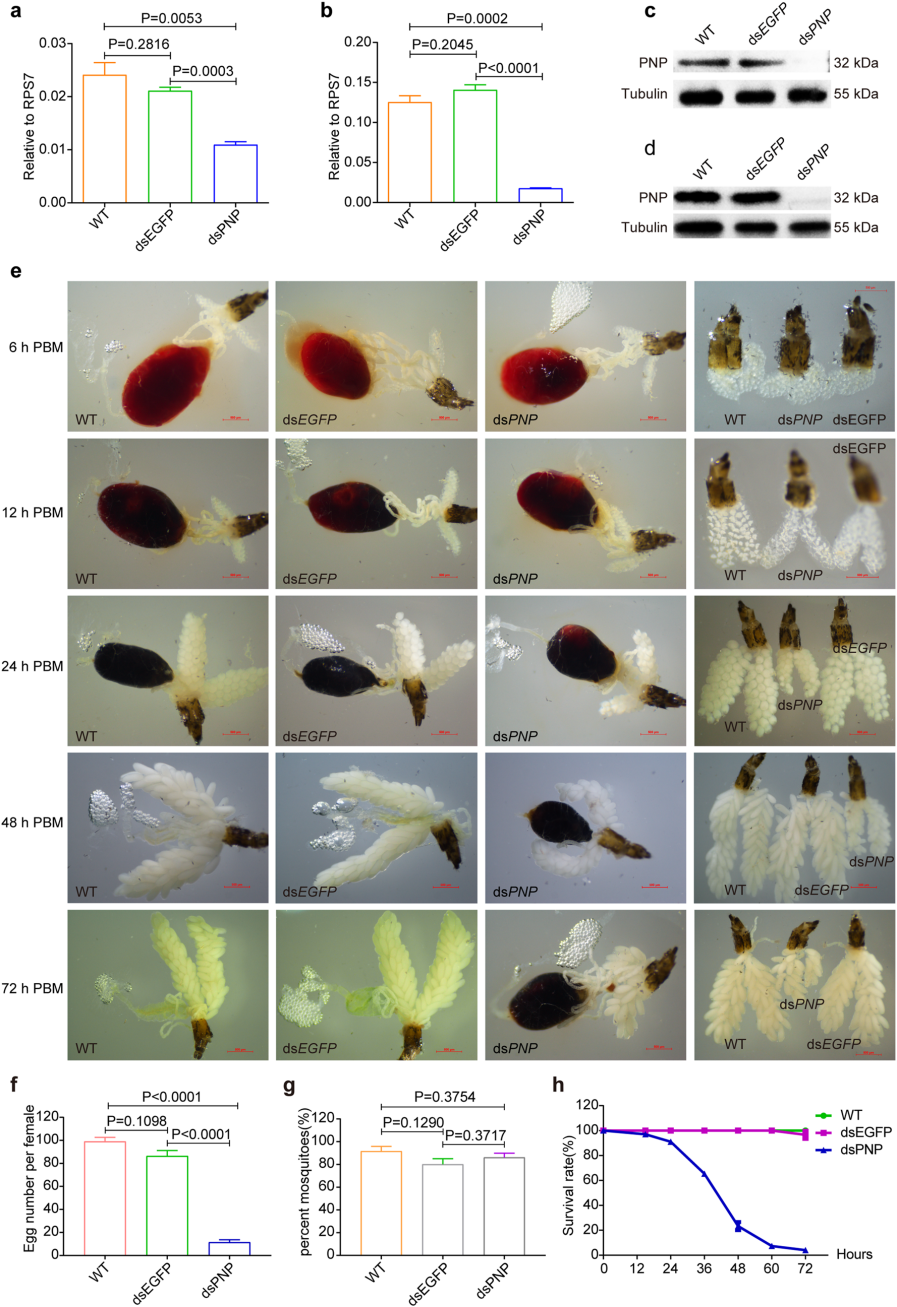
Abnormal phenotypes caused by *PNP* knockdown. **a** RT-qPCR results showing the knockdown of *PNP* at 72 h PE. **b** RT-qPCR results showing the knockdown of *PNP* at 6 h PMB. **c** Western blotting results showing the knockdown of *PNP* at 72 h PE. **d** Western blotting results showing the knockdown of *PNP* at 6 h PBM. **e** Phenotypes caused by *PNP* RNA interference. **f** Statistics of egg laying (*N* = 10 females × 6 biological replicates). **g** Statistics of blood sucking rate (*N* = 40 females × 4 biological replicates). **h**. Statistics of survival rate at 72 h PBM (*N* = 60 females per group × 3 replicates). *RPS7* was used as the control for RT-qPCR, and ds*EGFP* was used as the control for RNA interference. Error bars depict ± SEM of three independent experiments. Differences at *P* < 0.05 were considered to be statistically significant, and differences at *P* < 0.01 were considered to be highly statistically significant. dsEGFP, Double-stranded RNA of enhanced green fluorescent protein; dsPNP, double-stranded RNA of purine nucleoside phosphorylase; PBM, post blood meal; PE, posteclosion; RT-qPCR, real-time quantitative PCR; RPS7, ribosomal protein S7; SEM, standard error of the mean; WT, wild type

In order to reveal the function of *PNP* in *Ae. aegypti* mosquitoes, we injected the dsRNA of *PNP* at about 12–20 h PE and confirmed its downregulation by RT-qPCR and western blotting assays (Fig. 6a–d). *PNP* silencing did not affect sugar absorption or blood intake, and no evident alterations in morphology were observed at 6 h PBM in ds*PNP*-injected females in comparison with the control samples. However, abnormal phenotypes in blood digestion and ovary growth emerged from 12 h PBM onwards in these ds*PNP*-injected females (Fig. 6e). More specifically, ds*PNP*-injected mosquitoes displayed slower blood digestion and smaller ovaries compared with ds*EGFP*-injected females and wild-type controls. The differences in blood color and ovary size were more obvious at 24 h PBM. When the mosquitoes were dissected at 48 and 72 h PBM, in the mosquitoes in the two control groups, almost no blood was remaining in the midgut and the ovaries showed normal development; in comparison, in mosquitoes in the ds*PNP* group, at least half of the ingested blood was undigested and ovary development was stagnant. Notably, *PNP*-depleted mosquitoes began to die at 15 h PBM, half of them had died within 48 h PBM and almost all of them had died within 72 h PBM (Fig. 6f). Finally an average 100-fold oviposition reduction was observed in ds*PNP*-injected females in comparison with eggs collected from WT and ds*EGFP*-injected females (Fig. 6g, h).

### PNP downregulation affected the signaling pathways in oocyte development

**Fig. 7:**
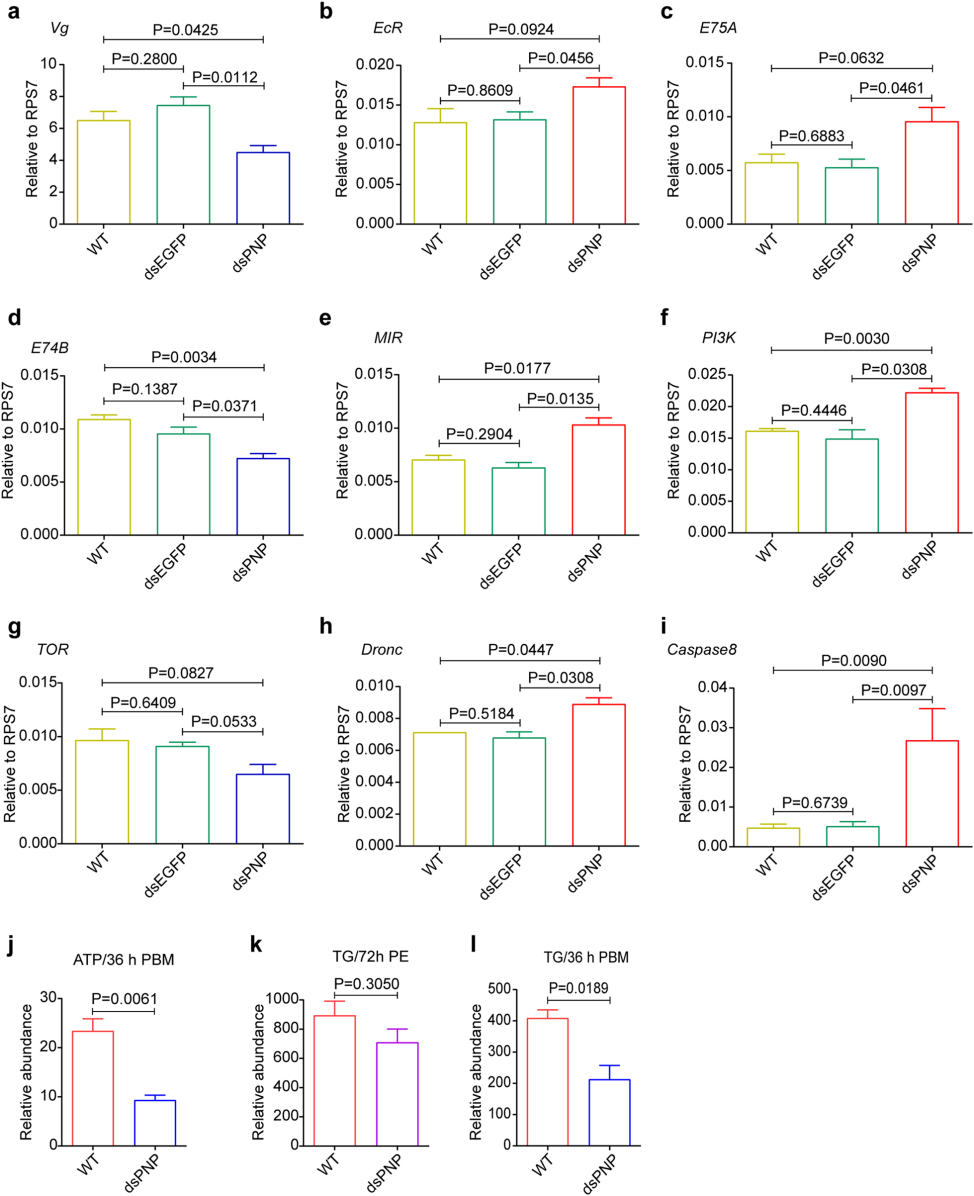
Knockdown of *PNP* in mosquitoes changes the expression of some signaling pathway genes and affects energy metabolism. Depicted are expression of the following. **a** Vitellogenin gene (*Vg*). **b** Ecdysteroid receptor gene (*EcR*). **c** Ecdysone-induced protein 75A gene (*E75A*). **d** Ecdysone-induced protein 74B gene (*E74B*). **e** Mosquito insulin receptor gene (*MIR*). **f** Phosphoinositide 3-kinase gene (*PI3K*). **g** Target of rapamycin gene (*TOR*). **h** Death regulator Nedd2-like caspase gene (*Dronc*). **i** Autophagy related 8 gene (*ATG8*). **j** ATP level at 36 h PBM. **k** TG level at 72 h PE without a blood meal.** l** TG level at 36 h PBM. Data are presented as the mean ± SEM of 3 independent experiments. Statistical comparisons between groups were performed using a one-way ANOVA followed by an unpaired Student’s t-test. Differences at *P* < 0.05 were considered to be statistically significant, and differences at *P* < 0.01 were considered to be highly statistically significant. ANOVA, Analysis of variance; dsEGFP, Double-stranded RNA of enhanced green fluorescent protein; dsPNP, double-stranded RNA of purine nucleoside phosphorylase; RPS7, ribosomal protein S7; TG, triglyceride; WT wild type

After *PNP* downregulation, mosquito blood digestion and ovary development were seriously inhibited. To understand the potential molecular mechanism of *PNP* in these physiological processes, we investigated the transcript level of a number of genes known to act in the signaling pathways involved in oocyte development (Fig. 7). Ovary development depends on the synthesis of vitellogenin (Vg) protein in the fat body [31]. We found that the *Vg* expression level was significantly downregulated at 24 h PBM in the *PNP*-depleted mosquitoes (Fig. 7a). The synthesis of Vg is mainly associated with juvenile hormone signaling, ecdysone, insulin-like peptide and amino acid nutrient signaling [32–35]. *PNP* is highly expressed in blood-fed mosquitoes and, therefore, *PNP* might function in mosquitoes after blood absorption; in comparison, the juvenile hormone signaling pathway generally affects the development of the ovary before blood absorption. Examination of the transcript levels of some key genes in ecdysone signaling, insulin-like peptide signaling and amino acid nutrient signaling at 24 h PBM revealed that *EcR* and *E75A* were upregulated and *E74B* was downregulated (Fig. 7b–d), the insulin-like peptide pathway genes *MIR* (mosquito insulin receptor) and *PI3K* (phosphatidylinositol 3-kinase) were upregulated (Fig. 7e, f), but ILP3 (insulin-like peptide 3) and *FoxO* (forkhead box O) were unchanged (data not shown). *TOR* (target of rapamycin), a key gene of the amino acid nutrient signaling pathway, showed no significant change in expression level (Fig. 7g). Important genes in the apoptotic pathway, namely *Dronc* and *caspase8*, were significantly upregulated (Fig. 7h, i), indicating that the apoptotic pathway may be activated. Indeed, a large number of cells showed programmed death, resulting in massive death of female mosquitoes at 36 h PBM.

*PNP*-depleted mosquitoes began to lose the ability to fly at 15 h PBM and ultimately died within 72 h PBM. PNP is a conserved key enzyme involved in the purine degradation and salvage pathway, which can help to synthesize ATP. We hypothesized that downregulation of *PNP* might also affect the metabolism of material and energy in fat bodies. As expected, the ATP content of the *PNP*-depleted female mosquitoes was far lower than that of WT mosquitoes (Fig. 7j). Lipids are stored in the fat body, and the use of lipid for basic metabolism requires a continuous mobilization of triglycerides (TG) from the fat body to other tissues. The fat bodies of the ds*PNP*- and ds*GEFP*-injected groups were harvested at both 72 h PE and 36 h PBM to determine the TG content. The results showed that no significant change in TG content had occurred before blood feeding (Fig. 7k) but that TG content was significantly decreased in the ds*PNP* mosquitoes at 36 h PBM (Fig. 7l). We therefore concluded that *PNP* downregulation could disturb utilization of nutrients and transformation of energy in mosquitoes, block the nutritional signaling pathway in the fat body and hinder ovarian development.

## Discussion

Our previous studies showed that when the mosquito- and midgut-specific miR-1174 is silenced, mosquitoes cannot absorb sugar and blood as normal, ovary development is stunted and, as a result, the mosquito reproduction rate is reduced; all of these abnormal phenotypes can be partially restored by RNAi knockdown of its target gene *SHMT* [7]. However, *SHMT* knockdown does not inhibit the absorption of both sugar and blood; it only inhibits the digestion, absorption and utilization of blood, thus affecting ovary development and reducing the oviposition rate [8]. Since *SHMT* is negatively regulated by miR-1174, logically, if miR-1174 only targeted *SHMT*, the abnormal phenotypes caused by miR-1174 depletion should be consistent with the abnormal phenotypes observed after *SHMT* overexpression, whereas the abnormal phenotypes after *SHMT* RNAi should be consistent with the phenotypes caused by miR-1174 overexpression. However, so far, it is not clear whether *SHMT* is the only target gene of miR-1174 in mosquitoes. We found that miR-1174 was only highly expressed in the midgut of mosquitoes and that it was not expressed or showed only trace expression in other tissues. On the contrary, there was no or only trace expression of *SHMT* in the midgut, but it was highly expressed in the fat body outside the midgut. Since miR-1174 and *SHMT* are specifically expressed in different tissues, other interaction modes or signaling pathways may exist between them. Therefore, in this study, at a time point when the adult mosquitoes have completely matured and fully recovered from the injection wound, we collected Ant-1174-injected mosquitoes and controls for proteomic and metabolomic detection, in the expectation of screening for DEPs and DEMs. To further reveal the molecular mechanism of this miRNA as a key regulatory molecule in blood-sucking mosquitoes, we functionally analyzed the signaling pathways associated with these DEPs and DEMs.

Both GO enrichment analysis and KEGG pathway enrichment analysis provided evidence that miR-1174 mainly acts in the metabolic pathways for amino acids, nucleotides, fatty acids and sugar. It should be noted that although these DEPs were detected after miR-1174 silencing, their differential expression is not necessarily directly caused by miR-1174 depletion. Generally, only the target genes are directly regulated by this miRNA, while the differential expression of those non-target genes are much more likely to be indirectly regulated by this miRNA. In this context, among the upregulated proteins, only *SPI27A* (AAEL014079), *MCT12* (AAEL002412) and *UC* (AAEL015019) were confirmed to be the target genes of miR-1174 (Fig. 3). RNAi knockdown of these three target genes did not cause any abnormal phenotypes in the experimental mosquitoes. One possible explanation for this result is that the RNAi knockdown was not enough to completely prevent the expression of the corresponding proteins, which are probably still functioning in mosquitoes. Admittedly, therefore, stronger evidence through knockout and overexpression might be required to determine the biological functions of these target genes in mosquitoes.

To reveal the biological roles of other, more miR-1174-responsive genes, we knocked them down one by one using RNAi technology and found that abnormal phenotypes were only observed in *PNP*-depleted mosquitoes after blood feeding. *PNP* depletion did not affect normal blood intake, but blood digestion and ovary development were blocked, and the life span of mosquitoes was shortened. *PNP* was highly expressed in the fat body, so we assumed that its downregulation would directly affect nutrient metabolism in the fat body. Vitellogenin protein (Vg) is synthesized in the fat body and is related to juvenile hormone signaling, ecdysone signaling, insulin-like peptide signaling and amino acid nutrient signaling [35–37]. RT-qPCR results showed that *PNP* depletion led to a significant downregulation of *Vg* and that ecdysone signaling pathway and insulin-like peptide signaling pathway related to ovarian development were also affected. However it remains unknown about whether and how *PNP* regulates the synthesis and expression of vitellogenin through these hormonal pathway signals.

*PNP* functions in pathways of purine, pyrimidine and niacin-nicotinamide metabolism; notably, the niacin-nicotinamide metabolism pathway can produce NADH. Further oxidative phosphorylation of NADH can produce a large amount of ATP to maintain life activities [38, 39]. The expression of the PNP protein was significantly upregulated after the downregulation of miR-1174, suggesting that miR-1174 may directly or indirectly target *PNP*. However, both the predicted results and the results of the dual-luciferase reporter assays indicated that *PNP* was not a direct target gene of miR-1174. Therefore, miR-1174 is very likely to regulate *PNP* indirectly through other genes or other signaling pathways. Disorder in glycine, serine and threonine metabolism affects purine metabolism and reduces AMP and ADP, which will lead to a decrease of ATP [40]. We found that depletion of miR-1174 affected the glycine, serine and threonine metabolic pathways, as well as the purine and pyrimidine metabolic pathways, suggesting that miR-1174 regulates blood digestion and ovary development of *Ae. aegypti* by participating in energy and nucleotide metabolism.

*PNP* reversibly phosphorylates purine nucleosides into purine bases and ribose-1-phosphate in the salvage pathways of purines, and *PNP* deficiency leads to accumulation of purine nucleosides [41]. In mammals, *PNP* absence leads to a significant increase in the affinity of deoxycytidine kinase (dCK) to deoxycytidine, which means a significant amount deoxycytidine triphosphate (dGTP) will be synthesized, and excessive dGTP will break the balance of deoxyribonucleotides, affect DNA synthesis, and induce T cell apoptosis [42]. Deficiency of *PNP* causes adenosine accumulation and apoptosis of tumor cells [43]. Key genes in the apoptotic pathway were detected after *PNP* knockdown, and both the *Dronc* and *caspase8* genes were significantly upregulated. *caspase8* is an important gene for initiating apoptosis, and its activation can activate other known caspase genes [44]. *PNP*-depleted mosquitoes showed a significant downregulation of ATP and TG content *in vivo*. These mosquitoes died within 48 h after blood inhalation, possibly due to the lack of ATP and TG which activates the apoptotic pathway, leading to abnormal programmed death of a large number of cells.

*PNP* is slightly expressed or even not expressed at all in the midgut and ovary, and highly expressed in the head, fat body and other residual tissues. However, when it was knocked down, the midgut function and ovary development were seriously affected. *PNP* silencing might first exert an effect on the fat body, and the responding signals then be transmitted to the midgut. This would then affect the expression of digestive enzymes, leading to malfunctions in blood digestion and nutrient absorption and utilization, so that there are not enough nutrients to synthesize vitellogenin precursors, ultimately resulting in abnormal ovary development. Although *PNP* was significantly upregulated in the miR-1174-depleted mosquitoes, we did not find any binding sites of miR-1174 within its 5’-UTR, CDS or 3’UTR. This implies that *PNP* is indirectly regulated by miR-1174, which might regulate important life activities such as sugar absorption, blood ingestion and digestion, ovary development and even life span through synergistically regulating multiple signaling pathways by multiple target genes. Although we have identified DEPs in this study, further studies are still needed to determine which of these DEPs are directly regulated by miR-1174 and which are indirectly regulated, and which of these genes are responsible for different types of abnormal phenotypes at different response levels after downregulation of miR-1174.

## Conclusions

This study investigated the molecular mechanism of the mosquito- and midgut-specific miR-1174 in *Ae.aegypti* mosquitoes. By using proteomics and metabolomics techniques, we identified the DEPs and DEMs in miR-11174-depleted mosquitoes. Their functional analysis showed that miR-1174 depletion affected pathways of amino acid, nucleotide, fatty acid and glucose metabolism. RNAi knockdown was performed to study further the roles of some DEPs. Specifically, we targeted purine nucleoside phosphorylase (*PNP*, AAEL002269), an upregulated gene which is not directly targeted by miR-1174, but has important functions in mosquitoes. When *PNP* was downregulated, blood digestion was inhibited, ovary development was hindered and the life span of mosquitoes was shortened. Downregulation of *PNP* affects hormone signaling pathways, apoptosis pathways and some nutrient metabolic pathways, suggesting that *PNP* may regulate these signaling pathways to control blood digestion and ovary development. Taken together, our findings revealed the molecular changes caused by miR-1174 depletion at the proteome and metabolome levels, and established the biological roles of some DEPs. As such, they provide valuable insights into the roles of a lineage-specific miRNA in the regulatory pathways of blood digestion and nutrient signaling.

### Supplementary Information

**Additional file 1: Table S1**. The original data extracted from 6 experimental samples.

**Additional file 2: Table S2**. The retained 5875 proteins after pretreatment.

**Additional file 3: Table S3**. Differentially expressed proteins screened according to the described criteria.

**Additional file 4: Table S4**. The retained 5714 peaks in POS mode after pretreatment.

**Additional file 5: Table S5**. The retained 5609 peaks in NEG mode after pretreatment.

**Additional file 6: Table S6**. The retained 11270 peaks in the combined POS and NEG modes after pretreatment.

**Additional file 7: Table S7**. Primers sequences used in this study.

**Additional file 8: Table S8**. All of the differentially expressed proteins (DEPs) after differential screening.

**Additional file 9: Table S9**. All of the differential metabolites(DEMs) after differential screening.

**Additional file 10: Table S10**. Data matrix for the hierarchical clustering of DEMs.

**Additional file 11: Table S11**. Classification information of DEMs. Red here presents the upregulated DEMs in each super class shown in Fig.2F; blue represents the downregulated DEMs in each super class shown in Fig.2F.

**Additional file 12: Table S12**. Pathway enrichment analysis of these DEMs.

## Data Availability

All data supporting the findings of this study are available within the article and its Additional files.

## Abbreviations

Ant: Antisense oligonucleotides (antagomirs)
DEPs: Differentially expressed proteins
DEMs: Differentially expressed metabolites
GO: Gene Ontology
KEGG: Kyoto Encyclopedia of Genes and Genomes
miR-1174: microRNA-1174
miRNA: MicroRNA
PBM: Post blood meal
PE: Posteclosion
PIJ: Post-injection
*PNP*: Purine nucleoside phosphorylase gene
RNAi: RNA interference
RT-qPCR: Real-time quantitative PCR
*SHMT*: Serine hydroxymethyltransferase gene
TMT: Tandem mass tag
3’-UTR: 3′-untranslated region
3′-RACE: 3‘-rapid amplification of cDNA end
dsEGFP: Double-stranded RNA of enhanced green fluorescent protein
dsPNP: Double-stranded RNA of purine nucleoside phosphorylase
WT: Wild type
RPS7: Ribosomal protein S7
SEM: Standard error of the mean
FB: Fat body
HD: head
LO: leftover
Mg: midgut
